# Supplementary material for: The Impact of Social Distancing Due to the COVID-19 Pandemic on People with Dementia, Family Carers and Healthcare Professionals: A Qualitative Study
Source: Int J Environ Res Public Health. 2022 Jan 4;19(1):519. doi: 10.3390/ijerph19010519 (PMC8744737; doi:10.3390/ijerph19010519)
Supplement: Supplementary file 1 [file ijerph-19-00519-s001.zip › ijerph-1494802-supplementary.pdf]

The impact of social distancing due to the COVID-19 pandemic on people with dementia, family carers and healthcare professionals: a qualitative study

Supplement I: Topic list interview of the ISOLATE study

**Demographic data**
1. Do you provide care in the residential setting or home care or are you a family carer?
2. How old are you?
3. What is your gender?
4. What is your ethnicity?
5. What is your highest level of education?
6. How many years of experience do you have as a care provider?
7. What is your relationship to the person with dementia that you care for as an informal carer? *Continue with questions about the person with dementia (such as severity and type of dementia, when did it start developing, etc.)*
8. On average, how many hours a week did you spend on caring for [people with dementia / your relative] during the COVID-19 pandemic? *If family carer: Since when have you been their carer?*
9. Is that more or less than you were used to in comparison with the period before the COVID-19 pandemic? What was the reason for this difference, if any?
**Semi-structured interview**
**How did you experience caring for your relative/people with dementia before the pandemic?** (a) first address how the person(s) with dementia experience(s) the pandemic (b) how does the interviewee experience the pandemic as it relates to the care for the person(s) with dementia *Orientation to gain a picture of how the participant views the care without the influence of the COVID-19 pandemic.*
**How did you experience the COVID-19 pandemic?** *Ask specifically about both the positive and the negative aspects of the pandemic for both the interviewee and the person(s) with dementia they care for.*
**In your opinion, what was the impact of social distancing during the COVID-19 pandemic on (the behaviour of) [people with dementia / your relative]?**

Specifically ask about the influence of social distancing on:<br><br>• *Health care*<br>• *Well-being (physical, psychological, and social)*<br>• *Social contacts*<br>*and the relationship between these three themes*<br>*Remember to also ask about the different periods (total closure, restrictions)*<br>*How did the behaviour develop over time?*
**What are your biggest concerns regarding social distancing and people with dementia?**<br><br>*Ask specifically about concerns regarding:*<br><br>• *Care*<br>• *Well-being (physical, psychological, and social)*<br>• *Social contacts*
**What do you think is needed in the future to reduce your concerns regarding social distancing and people with dementia?**<br><br>*Try to refer back to the positive experiences mentioned earlier.*<br><br>*And specifically ask about concerns regarding:*<br><br>• *Care*<br>• *Well-being (physical, psychological, and social)*<br>• *Loneliness*
**What is your opinion on the current healthcare policy for people with dementia during the COVID-19 pandemic?** *Focus on "What would you do?"*<br><br>*Ask specifically about concerns regarding:*<br><br>• *Care*<br>• *Well-being (physical, psychological, and social)*<br>• *Social contact (and loneliness)*
**What do you think needs to be improved about the healthcare policy to safeguard the care and well-being of people with dementia during any future pandemic?**<br><br>*Ask specifically about policies related to:*<br><br>• *Care*<br>• *Well-being (physical, psychological, and social)*

• *Loneliness*<br>*If relevant, ask about policies for supporting family carers and healthcare professionals.*
**What are your greatest concerns regarding social distancing and your own situation?**<br><br>*Ask specifically about concerns regarding:*<br><br>• *Care*<br>• *Well-being (physical, psychological, and social)*<br>• *Social contacts (also ask about the loneliness of people involved)*
**What do you think is needed in the future to reduce your concerns regarding social distancing and your own situation?**<br><br>*Try to refer back to the positive experiences mentioned earlier.*<br><br>*Ask specifically about concerns regarding:*<br><br>• *Care*<br>• *Well-being (physical, psychological, and social)*<br>• *Loneliness*
